# Regional Anesthesia With Paravertebral Blockade Is Associated With Improved Outcomes in Patients Undergoing Minithoracotomy Cardiac Surgery

**DOI:** 10.1177/15569845231190638

**Published:** 2023-08-10

**Authors:** Monica Hsieh, Diane Kim, Defen Peng, Travis Schisler, Richard C. Cook

**Affiliations:** 1 Department of Anesthesiology, Pharmacology, and Therapeutics, University of British Columbia, Vancouver, BC, Canada; 2 University of British Columbia, Vancouver, BC, Canada; 3 Centre for Cardiovascular Innovation, University of British Columbia, Vancouver, BC, Canada; 4 Division of Cardiovascular Surgery, University of British Columbia, Vancouver, BC, Canada

**Keywords:** minithoracotomy surgery, paravertebral blockade, regional analgesia

## Abstract

**Objective:**

Severe postoperative pain has been shown to affect many patients following minimally invasive cardiac surgeries (MICS). Multimodal pain management with regional anesthesia, particularly by delivery of local anesthetics using a paravertebral catheter (PVC), has been shown to reduce pain in operations involving thoracotomy incisions. However, few studies have reported high-quality safety and efficacy outcomes of PVCs following MICS.

**Methods:**

Patients who underwent MICS at Vancouver General Hospital between 2016 and 2019 (*N* = 123) were reviewed for perioperative opioid-narcotic use. Primary outcomes were postoperative opioid use and hospital length of stay (LOS). Statistical analyses were performed using univariate and multivariable regression models to determine independent risk factors.

**Results:**

A total of 54 patients received routine systemic analgesia (control), 53 patients received a paravertebral catheter (PVC), and 16 patients received another mode of regional analgesia (non-PVC). The mean hospital LOS was significantly different in patients in the PVC group at 5.8 ± 2.0 days versus 8.3 ± 7.1 days in the control and 6.6 ± 2.3 days in the non-PVC group (*P* = 0.033). The percentage of patients who did not require postoperative oxycodone was significantly higher in the PVC group (48.1%), compared with the control (24.5%) and non-PVC (37.5%; *P* = 0.043) groups.

**Conclusions:**

The administration of regional anesthesia using PVCs was associated with reduced need for opioids and a shorter LOS. The reduction in postoperative opioids may reduce the risk of potential opioid dependency in this population. Future studies should involve randomized controlled trials with systematic evaluation of pain scores to verify current study results.

## Graphical abstract

**Visual abstract:**
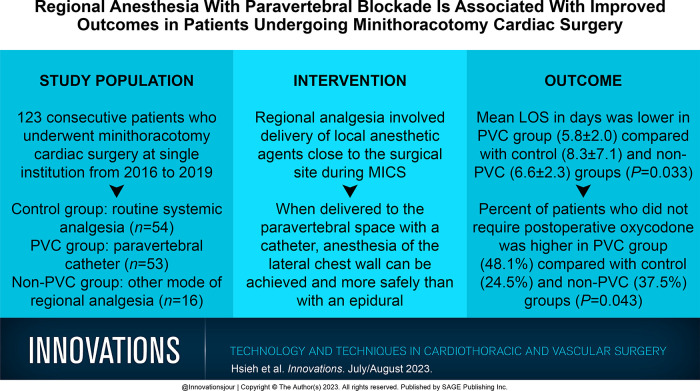

**Central Message**

One of the barriers to adoption of cardiac surgical procedures through a minithoracotomy incision is postoperative pain. In this study, we found that use of a paravertebral catheter significantly reduced the need for postoperative oral opioids and significantly reduced hospital LOS.

## Introduction

Cardiac surgery patients often face acute postoperative pain from surgical incisions of tissues and arteries, irritation of the pleura, intercostal nerve disruptions, and more.^1–5^ Inadequate pain management at this time can lead to chronic postoperative pain, which may severely impact the patient’s physiological functions and quality of life.^2,3,5^

Minimally invasive cardiac surgeries (MICS) have increasingly gained attention in clinical practice for their potentially faster recovery in line with the Enhanced Recovery After Surgery (ERAS) paradigm shift in surgical care. MICS through right minithoracotomies are currently performed for atrioventricular valve and aortic valve repairs or replacements, atrial septal defect closure (ASD), and ablations for atrial fibrillation.^6^

In addition to improving surgical outcomes, optimal pain management is essential to control patient therapeutic opioid consumption. The opioid epidemic has arisen as a major issue in North America, including the recent OxyContin® crisis, for which physician overprescription of narcotics may have exacerbated opioid dependencies.^7–10^ The need for innovative analgesic approaches is apparent to minimize not only postoperative pain-induced complications but also the consumption of opioid narcotics in patients undergoing cardiac operations.

Recent literature has explored the efficacy of multimodal therapies, including regional anesthesia, to strengthen ERAS for cardiac surgery patients.^11–15^ Paravertebral (PV) catheter-based regional anesthesia is regarded as one of the most promising options for providing regional anesthesia in cardiac surgical patients and has been studied in depth for patients undergoing thoracic surgical procedures.^14–17^ Nevertheless, further research is required to identify its efficacy and safety profile in patients undergoing MICS.

The purpose of this study was to determine whether patients undergoing MICS and who received different types of regional anesthesia during their perioperative period required less opioid narcotics and had better outcomes compared with patients who did not receive regional anesthesia.

## Methods

This was a retrospective nonrandomized review of 123 patients who underwent MICS at Vancouver General Hospital, a tertiary referral center in Vancouver, Canada, between January 2016 and December 2019. Specific procedures included mitral valve repair or replacement (MVR), tricuspid valve repair or replacement (TVR), and ASD. All operations were performed by a single surgeon.

### Inclusion and Exclusion Criteria

Inclusion criteria included all patients who had isolated MVR or TVR or isolated ASD via right minithoracotomy approach. To minimize confounding factors in our results, our study excluded redo procedures (i.e., prior sternotomy), combined procedures, and procedures in which intraoperative conversion to sternotomy was necessary.

### Patient Groups

Patients were identified as either control group (*n* = 54), paravertebral catheter (PVC) group (*n* = 53), or non-PVC group (*n* = 16) based on the type of perioperative regional anesthesia delivered. The non-PVC group included patients who received a single injection of local anesthetic as either a PV block (PVB) or erector spinae block (ESB) and patients who had an erector spinae catheter placed. The type of regional anesthesia used in any given patient was at the discretion of the attending anesthesiologist, and all of the options listed above were used during the study period.

### Anesthetic Protocol

The PVC group had a PVC inserted on the side of surgery before the induction of anesthesia. PVC placement was performed in the operating room prior to induction of anesthesia by an anesthesiologist trained in ultrasound or landmark-based placement with the patient in the sitting position. A linear ultrasound probe was placed in the parasagittal plane to find the transverse process at the fourth thoracic vertebrae. Local anesthetic was administered in the skin, and a 17 gauge Tuohy needle was inserted into the paravertebral space under ultrasound guidance. The desired endpoint of needle insertion was either direct needle visualization deep to the costotransverse ligament in the paravertebral space and/or depression of the parietal pleura upon injection of saline.

In patients with a body habitus that impeded adequate ultrasonography of the paravertebral space, a landmark-based technique was used. The paravertebral space was estimated 2.5 cm from the midline, and the Tuohy needle was advanced either cephalad or caudad off the transverse process to a successful endpoint as determined by the anesthesiologist. Following confirmation of successful needle placement, a stiff, non–wire-bound catheter was inserted into the paravertebral space to a depth of 3 to 5 cm. The catheter was bolused with 20 mL of 0.5% ropivacaine.

Standard anesthetic protocols included a balanced anesthetic induction with propofol, rocuronium, and an opioid: sufentanil, fentanyl, or hydromorphone. The maintenance of anesthesia was achieved with inhalational anesthesia, a sufentanil infusion at 0.2 to 0.5 µg/kg/h, or intermittent boluses of fentanyl or hydromorphone. An infusion of propofol or dexmedetomidine was used during cardiopulmonary bypass (CPB) and as sedation for transfer to the post–cardiac surgery unit. Most patients received a second bolus of 20 mL of 0.5% ropivacaine at the end of the procedure.

In the postoperative setting, patients in the catheter-based groups received a background infusion of 0.2% ropivacaine at 2 mL/h, with patient or nurse-controlled boluses of 8 mL as often as every hour. Catheters were typically removed on postoperative day 2 or 3 at the discretion of the anesthesiologist. Patients received acetaminophen 650 mg oral QID for 7 days as well as hydromorphone or oxycodone PRN for pain control. In patients without a contraindication, nonsteroidal anti-inflammatory drugs (NSAIDs) were also provided. The first-line oral NSAID used was naproxen 250 mg oral BID PRN.

### Outcomes

Data were collected through internal hospital records, following the institution’s research ethics guidelines. Basic patient demographics, time to extubation, intraoperative and postoperative opioid use, and hospital length of stay (LOS) were recorded. Time to extubation (min) was defined as the time between operating room exit to extubation time. For patients who were extubated in the operating room, time to extubation was calculated as the time from skin closure to operating room exit. Hospital LOS (days) was defined as the date of operation to date of discharge. Opioid usage was converted to morphine equivalents (micrograms) for comparison between anesthetics (hydromorphone, sufentanil, fentanyl, and oxycodone). Primary outcomes of the study included LOS and postoperative opioid use.

### Statistical Analysis

Description of the baseline cohort characteristics and intraoperative and postoperative comorbidities were presented for the respective groups. Discrete variables were summarized by frequencies and percentages. Percentages were calculated according to the number of patients. Continuous variables were summarized with mean ± standard deviation or median and interquartile interval (P25, P75). The 3 groups were compared using the chi-square test or Fisher’s exact test for categorical variables and Student’s *t* test or Wilcoxon rank-sum test for continuous variables, as appropriate.

To explore an appropriate statistical model, the distribution of the outcome was checked from the histogram and the description. Since LOS was overdispersed, a negative binomial regression model was used to explain the relationship between the covariates and hospital LOS. Due to the right-skewed distribution, the relationship between covariates and time to extubation or total postoperative opioid use was explored using nonlinear regression analysis based on logarithm transformation on the outcomes. Analysis of oxycodone use required logistic regression. Univariate and multivariable regression analyses were used to explore potential risk factors.

The variables considered to be adjusted in the regression models were as follows: demographic factors (age, sex, body mass index [BMI]), preoperative risk factors (urgency of surgery, New York Heart Association [NYHA] class, left ventricular ejection fraction [LVEF], atrial fibrillation (Afib), smoking, hypertension), intraoperative (total surgery time, CPB time, cross-clamp [XC] time), and postoperative comorbidities (estimated glomerular filtration rate, postoperative Afib/arrhythmia).

All of the model selections were developed by incorporating all variables using multiple model selection procedures (both stepwise selection and backward elimination techniques) with statistical significance of inclusion at *P* ≤ 0.20 and exclusion at *P* < 0.05. All reported *P* values are two-tailed under the conventional 5% level of significance, and all statistical analyses were performed with SAS software version 9.4 (SAS Institute, Cary, NC, USA).

## Results

### Patient Characteristics, Preoperative Risk Factors, and Comorbidities

Patient demographics in regard to sex and BMI were comparable between the control, non-PVC, and PVC groups. However, age was significantly different (*P* = 0.021). Therefore, subsequent outcome analyses have been adjusted accordingly. Of the preoperative risk factors studied, hypertension varied significantly, with about 50% of the patients in the control and non-PVC groups and only 26.4% in the PVC cohort known to have the condition (*P* = 0.021; Table 1).

**Table 1.** Patient Characteristics and Preoperative Risk Factors.

	All (*N* = 123)	Control (*n* = 54)	Non-PVC (*n* = 16)	PVC (*n* = 53)	*P* value
Age, years	60.3 ± 15.9	64.5 ± 14.2	53.4 ± 15.2	58.1 ± 16.9	0.021
Male sex	76 (61.8)	32 (59.3)	9 (56.3)	35 (66.0)	0.68
Overweight BMI	60 (48.8)	26 (48.1)	9 (56.3)	25 (47.2)	0.81
Urgent surgery	14 (11.4)	6 (11.1)	3 (18.8)	5 (9.4)	0.57
NYHA class III/IV	37 (30.1)	20 (37)	6 (37.5)	11 (20.8)	0.15
LVEF ≤60%	33 (27.0)	14 (25.9)	2 (13.3)	17 (32.1)	0.38
Hypertension	50 (40.7)	28 (51.9)	8 (50.0)	14 (26.4)	0.021
Current/former smoker	29 (23.8)	14 (26.4)	5 (31.3)	10 (18.9)	0.50
Atrial fibrillation	13 (10.8)	4 (7.8)	2 (12.5)	7 (13.2)	0.65
Renal dysfunction	5 (4.1)	3 (5.7)	0	2 (3.8)	0.99

Abbreviations: BMI, body mass index; LVEF, left ventricular ejection fraction; NYHA, New York Heart Association; PVC, paravertebral catheter.

Data are reported as mean ± SD or *n* (%).

Intraoperative characteristics that were found to be statistically different were total surgery, CPB, and XC times, which were lowest in the PVC group (*P* = 0.003, *P* < 0.001, and *P* < 0.001, respectively). Postoperative comorbidities were comparable for all 3 groups (Table 2). There were no perioperative deaths or strokes.

**Table 2.** Intraoperative and Postoperative Comorbidities.

	All (*N* = 123)	Control (*n* = 54)	Non-PVC (*n* = 16)	PVC (*n* = 53)	*P* value
Total surgery time, hours	4.8 ± 1.1	5.1 ± 1.2	4.8 ± 1.0	4.4 ± 1.0	0.003
CPB pump time, minutes	168.6 ± 44.6	186.6 ± 44.8	164.7 ± 51.6	152.5 ± 35.6	<0.001
XC time, minutes	120.4 ± 37.2	138.7 ± 35.7	112.1 ± 38.2	106.0 ± 31.3	<0.001
eGFR, mL/minute	87.2 ± 25.2	82.7 ± 25.6	81.6 ± 16.1	93.4 ± 25.9	0.06
CVA	1 (0.8)	1 (1.9)	0	0	0.99
Postoperative Afib/arrhythmia	46 (37.7)	15 (28.3)	7 (43.8)	24 (45.3)	0.17
Wound infection	1 (0.8)	0	0	1 (1.9)	0.99

Abbreviations: Afib, atrial fibrillation; CPB, cardiopulmonary bypass; CVA, cerebrovascular accident; eGFR, estimated glomerular filtration rate; PVC, paravertebral catheter; XC, cross-clamp.

Data are reported as mean ± SD or *n* (%).

### Time to Extubation

Median times to extubation in the PVC, control, and non-PVC groups were 313 (230, 390) min, 397 (242.5, 570) min, and 366 (197, 740) min, respectively, which was not significantly different overall (*P* = 0.09; Table 3). However, when comparing the PVC group to the control group only, unadjusted nonlinear regression analysis revealed a significantly lower extubation time in the PVC group compared with the control group (38.9% lower, *P* = 0.007). By univariate analysis, age, LVEF, Afib, smoking history, total surgery time, and CPB time were identified as potentially significant factors affecting time to extubation. After adjusting for the significant risk factors (age, LVEF, Afib, and smoking history) in multiple regression analysis, patients who had a PVC still had a significantly shorter time to extubation (45.3% lower, *P* < 0.001) than control group patients (Table 4).

**Table 3.** Summary of Outcomes.

	All (*N* = 123)	Control (*n* = 54)	Non-PVC (*n* = 16)	PVC (*n* = 53)	*P* value
Time to extubation, minutes	330.0 (232.5, 495.0)	397.0 (242.5, 570.0)	366.0 (197.0, 740.0)	313.0 (230.0, 390.0)	0.09
Hospital LOS, days	7.0 ± 5.0	8.3 ± 7.1	6.6 ± 2.3	5.8 ± 2.0	0.033
Total intraoperative opioids, morphine equivalents	144,005 (74,205, 209,750)	154,905 (90,005, 226,130)	113,700 (39,503, 211,575)	141,350 (74,000, 197,850)	0.49
Total postoperative opioids, morphine equivalents	127.5 (67.5, 253.5)	126.0 (75.0, 217.5)	211.1 (124.5, 283.1)	111.0 (43.1, 200.3)	0.09
Hydromorphone IV	0.0 (0.0, 6.0)	0.0 (0.0, 6.0)	0.0 (0.0, 11.3)	0.0 (0.0, 6.0)	0.75
Hydromorphone PO	45.0 (15.0, 90.0)	30.0 (15.0, 75.0)	93.8 (30.0, 200.6)	44.1 (15.0, 86.3)	0.12
Oxycodone PO	37.5 (0.0, 120.0)	60.0 (7.5, 157.5)	26.3 (0.0, 120.0)	7.5 (0.0, 67.5)	0.036
Postoperative oxycodone use	77 (63.6)	40 (75.5)	10 (62.5)	27 (51.9)	0.043

Abbreviations: LOS, length of stay; IV, intravenous, PO; per os; PVC, paravertebral catheter.

Data are reported as median (interquartile interval), mean ± SD, or *n* (%).

**Table 4.** Comparison of Outcomes Between Treatments.

	Univariate analysis		Adjusted for potential risk factors	
Outcomes	Estimate (95% CI)	*P* value	Estimate (95% CI)	*P* value
Time to extubation, minutes ^a^				
Non-PVC vs control	−0.22 (−0.76, 0.32)	0.43	−0.36 (−0.90, 0.18)	0.19
PVC vs control	−0.49 (−0.85, −0.13)	0.007	−0.60 (−0.95, −0.26)	<0.001
Hospital LOS, days ^b^				
Non-PVC vs control	−0.23 (−0.52, 0.05)	0.11	−0.07 (−0.29, 0.16)	0.55
PVC vs control	−0.36 (−0.55, −0.17)	<0.001	−0.21 (−0.36, −0.05)	0.010
Total dose of postoperative opioids, morphine equivalents ^c^				
Non-PVC vs control	0.40 (−0.15, 0.94)	0.15	0.11 (−0.39, 0.60)	0.67
PVC vs control	−0.25 (−0.63, 0.12)	0.18	−0.38 (−0.72, −0.05)	0.026
Postoperative oxycodone use ^d^				
Non-PVC vs control	0.54 (0.17, 1.78)	0.31	0.51 (0.15, 1.72)	0.28
PVC vs control	0.35 (0.15, 0.80)	0.013	0.35 (0.15, 0.81)	0.014

Abbreviations: CI, confidence interval; PVC, paravertebral catheter.

a Estimate: log of time to extubation; for PVC group compared with control group, (exp(−0.493) − 1) × 100 = 38.9% lower in univariate analysis, and (exp(−0.604) - 1) × 100 = 45.3% lower in multivariable analysis.

b Estimate: log of hospital LOS; for PVC group compared with control group, (exp(−0.36) − 1) × 100) = 30.2% shorter in univariate analysis, and (exp(−0.207) − 1) × 100) = 18.7% shorter in multivariable analysis.

c Estimate: log of total dose of postoperative opioids; for PVC group compared to control group, (exp(−0.254) − 1) × 100) = 22.4% less in univariate analysis, and (exp(−0.380) − 1) × 100) = 31.6% less in multivariable analysis.

d Estimate: odds ratio for postoperative oxycodone use.

### Hospital LOS

In the PVC group, the mean LOS was 5.8 ± 2.0 days. Patients in the non-PVC and control groups had mean LOS of 6.6 ± 2.3 days and 8.3 ± 7.1 days, respectively. These results were significantly different (*P* = 0.033; Table 3). In the univariate negative binomial regression model, the expected LOS for patients in the PVC group was about 30.2% shorter compared with patients in the control group (*P* < 0.001). By univariate analysis, age, urgent surgery, NYHA class III/IV, Afib, CPB time, XC time, and postoperative Afib/arrhythmia were identified as potentially significant factors affecting LOS. After adjusting for significant risk factors (age, NYHA class, and Afib) in multiple regression analysis, patients who had a PVC still had a significantly shorter LOS than control group patients, with the expected LOS for patients in the PVC group being about 18.7% shorter compared with patients in the control group (*P* = 0.010; Table 4).

### Intraoperative and Postoperative Opioid Consumption

Total intraoperative opioid consumption accounted for sufentanil, fentanyl, and hydromorphone. There was no significant difference in the total amount of morphine equivalent doses among the groups (*P* = 0.49; Table 3).

Postoperative opioid consumption accounted for oral oxycodone and both intravenous and oral hydromorphone (Table 3). In the nonlinear univariate regression analysis, total postoperative opioid use in the PVC group was about 22.4% less than that of the control group but was not statistically significant (*P* = 0.18). However, after adjusting for the significant risk factors of age and BMI in multiple regression analysis, total postoperative opioid use in the PVC group was about 31.6% less compared with the control group (*P* = 0.026; Table 4).

Of the postoperative opioids, oxycodone was the most important opioid used for patient-perceived pain, as the drug was given upon patient request only and was the preferred oral opioid used postoperatively on our site’s recovery ward. It was also the primary opioid prescribed at time of discharge. The results showed a significant association between oxycodone use and treatment groups (*P* = 0.043), such that almost half of the PVC cohort (48.1%) did not require oxycodone, followed by the non-PVC (37.5%) and control (24.5%) groups (Table 3). Even for patients who received oxycodone, the PVC group had a median oxycodone usage that was significantly lower at 7.5 (0, 67.5) µg, compared with 60.0 (7.5, 157.5) µg and 26.3 (0, 120.0) µg in the non-PVC and control groups, respectively (*P* = 0.036). Consumption of oxycodone was still lower in the PVC group compared with the control group in the univariate and multiple regression analyses when adjusted for age (odds ratio [OR] = 0.35, 95% confidence interval [CI]: 0.15 to 0.80, *P* = 0.013 and OR = 0.35, 95% CI: 0.15 to 0.81, *P* = 0.014, respectively; Table 4).

## Discussion

The key findings of this study were statistically significant reductions in (1) postoperative hospital LOS, (2) time to extubation, and (3) elective oxycodone consumption in patients who received PVC-based regional anesthesia compared with the control group.

Clinically, the observations of the health care staff looking after these patients postoperatively suggested that patients who received PVC-based regional anesthesia were the most comfortable in the cardiac surgery intensive care unit. Even so, the magnitude of the differences in outcomes between the PVC group and the control group was surprising to us and is perhaps a strong indication of the importance of pain control in recovery from MICS.

The findings of this study are also important because preferences for MICS are increasing. There are many reasons for this, including fewer patient limitations postoperatively and faster recovery.^18,19^ However, postoperative pain continues to be a significant source of morbidity with MICS, and there is empirical evidence that pain is an important variable impacting patient short-term and long-term recovery from cardiac surgeries.^1,3,5^

Regarding postoperative LOS, there are clearly many factors contributing to this outcome. At our institution, the surgeon is not involved in discharge planning, as established clinical pathways are used to guide patient discharges, with the final decision being made by a nurse practitioner or a family physician working on the recovery ward.

Previous studies have demonstrated that PVC-based regional anesthesia has a relatively low side effect profile, is associated with hemodynamic and respiratory stability and decreased LOS, with comparable or better pain control compared with the traditional thoracic epidural anesthesia (TEA).^11,15,20,21^ The PVC inserts into the paravertebral space, lateral to the vertebral columns as the nerves exit the spinal cord. In contrast, neuraxial TEA directly invades the epidural space and inevitably increases the risk of major complications such as spinal hematoma and abscess, dural puncture, and nerve injuries.^1,13,14^ Unlike TEA, PVC-based regional anesthesia can spare normal respiratory and sympathetic functions on the contralateral side with unilateral blocks, thereby reducing the incidence of hypotension, pulmonary complications, urinary retention, and other complications seen with TEA.^12,13^ Systemic anticoagulation with intravenous heparin can also be used safely with PVCs.

Our local observations were also our rationale for including a “non-PVC” group in our study. Early in our experience, the optimal method of delivering regional anesthesia was not clearly established at our center nor in the literature. There are several other ways of providing regional anesthesia to the chest wall, including intercostal nerve blocks, intrapleural blocks, serratus anterior plane (SAP) block, and ESB.

The intercostal nerve blocks are arguably easiest to perform and involve injection of local anesthetic into the intercostal muscles between the relevant ribs.^22^ When compared with TEA, intercostal nerve blocks were inferior for pain relief.^23^ This was the only method of regional anesthesia used at our center for many years and was felt to be minimally effective at reducing postoperative pain. However, more recently, some centers have used intercostal nerve cryoablation, where high-pressure CO_2_ or nitrous oxide are used to cool the intercostal nerves to −50 to −70 °C, with good pain control, decreased hospital LOS, and postoperative opioid consumption.^24^

For intrapleural blocks, the local anesthetic is inserted via a catheter between the visceral and parietal pleura. When compared with TEA, intrapleural blocks have been shown to provide superior pain relief; however, careful placement and manipulation of the catheter by the surgical team was required.^25,26^

In the case of SAP blocks, the local anesthetic is injected between the serratus anterior and latissimus dorsi muscle. A comparison between SAP and paravertebral blocks for minimally invasive coronary bypass surgery demonstrated that patients receiving the SAP block had higher postoperative opioid consumption.^27^

ESBs are performed by inserting the local anesthetic ventral to the erector spinae muscle. Superior analgesia has been observed when comparing ESBs to intercostal blocks.^28^

From our anecdotal experience with several of these strategies, including PVB, ESB, and ESC, it soon became clinically evident to the anesthesiologists and nurses that those who had a properly functioning PVC had the best pain control. Therefore, PVC-based regional anesthesia became the preferred approach over the course of 2018.

Despite its theoretically proposed benefits, current literature on the effects of PVC in MICS patients is limited. Many studies show small total sample sizes (*N* < 100), lack a control group, have limited parameters to determine efficacy, or are not randomized.

Although this is a single-center retrospective review of the application of regional anesthesia techniques in patients undergoing MICS, it represents a continuous series of patients who had surgery in a contemporary time span. Furthermore, this study is one of the most recent reports with a large control group and a comparatively larger sample size that examines both intraoperative and postoperative parameters.

Pain scores were not systematically collected at consistent time points, and therefore, the quality of pain score data was not optimal. However, anecdotally, patients were more comfortable with a PVC, and this was reflected in their significantly decreased postoperative oral oxycodone use, which is an important clinical indication of improved patient-perceived pain. In fact, it has become the standard of practice to place PVCs in patients undergoing MICS at our institution, despite the added procedural time, because the observed reduction in postoperative pain was clearly evident. These results are despite the fact that not all patients in the PVC group had a catheter that worked well. There is still heterogeneity among the anesthesiologists in terms of their ability to place properly functioning catheters despite the use of ultrasound, which has been the standard of practice since approximately 2010.

With regard to time to extubation, it was not surprising to us to witness faster times to endotracheal extubation in the PVC group, as it has been demonstrated that the use of multimodal analgesia does contribute to earlier extubation times.^29,30^ Opioid-sparing anesthesia techniques, including regional or neuraxial techniques,^31–33^ are now components of many ERAS protocols and have been proven to decrease time to endotracheal extubation.

In addition to postoperative pain, the opioid crisis is an ongoing public health care problem that has contributed to an epidemic of drug-related overdose deaths.^34^ Sixty-seven percent of the overdose-related deaths in America recorded in 2018 were caused by prescription or illicit opioids like OxyContin.^8,35^ In Canada, accidental injuries are the third leading cause of mortality, which has largely been attributable to apparent opioid toxicity deaths in 2017.^9,36^

Identifiable factors to this epidemic include physician overprescription of opioids and the emergence of illicit synthetic opioids such as fentanyl and carfentanil.^7,9^ Since PVC-based regional anesthesia was associated with significantly fewer patients using postoperative oxycodone, this may translate into fewer patients being at risk for developing opioid dependency after cardiac surgery.

## Conclusions

In this retrospective comparison of a series of consecutive patients who underwent MICS, the group of patients who had a PVC placed preoperatively appeared to have benefitted in 3 important ways: (1) less pain, as suggested by a decreased need for oral opioids in the postoperative period; (2) quicker recovery from surgery, as demonstrated by shorter time to extubation and a dramatically reduced postoperative LOS; and (3) a theoretically lower risk of opioid dependency, given the 50% reduction in the number of patients who required oxycodone in the postoperative period. Given the natural limitations of retrospective chart reviews, further prospective, randomized studies should be performed to verify these outcome benefits. Systematic recording of pain scores at specific time intervals may enhance the anecdotal evidence of patient pain management.
